# Using Metaphors to Understand Suffering in COVID-19 Survivors: A Two Time-Point Observational Follow-Up Study

**DOI:** 10.3390/ijerph20021390

**Published:** 2023-01-12

**Authors:** Alvisa Palese, Erica Visintini, Valentina Bressan, Federico Fonda, Stefania Chiappinotto, Luca Grassetti, Maddalena Peghin, Carlo Tascini, Matteo Balestrieri, Marco Colizzi

**Affiliations:** 1 School of Nursing, Department of Medicine (DAME), University of Udine, 33100 Udine, Italy; 2 Department of Economics and Statistics (DIES), University of Udine, 33100 Udine, Italy; 3 Infectious and Tropical Diseases Unit, Department of Medicine and Surgery, University of Insubria-ASST-Sette Laghi, 33100 Varese, Italy; 4 Infectious Diseases Division, Department of Medicine (DAME), University of Udine, 33100 Udine, Italy; 5 Unit of Psychiatry, Department of Medicine (DAME), University of Udine, 33100 Udine, Italy; 6 Department of Psychosis Studies, Institute of Psychiatry, Psychology and Neuroscience, King’s College London, London SE5 8AF, UK

**Keywords:** COVID-19, coronavirus disease 2019, follow-up, lived experience, qualitative study, longitudinal study, metaphors

## Abstract

Accumulating evidence indicates that the COVID-19 pandemic carries risks to psychological health and represents a collective traumatic experience with consequences at the social, economic, and health levels. The primary aim of this study was to collect ongoing COVID-19 survivors’ pandemic-related experiences as expressed through the use of metaphors; the secondary aim was to explore socio-demographic variables associated with the metaphor orientation as negative, positive or neutral. An observational follow-up survey was conducted and reported according to the STROBE guidelines. Patients ≥ 18 years, who were treated for COVID-19 during the first wave (March/April 2020) and who were willing to participate in a telephone interview were involved and asked to summarize their COVID-19 experience as lived up to 6 and 12 months in a metaphor. A total of 339 patients participated in the first (6 months) and second (12 months) data collection. Patients were mainly female (51.9%), with an average age of 52.9 years (confidence interval, CI 95% 51.2–54.6). At 6 months, most participants (214; 63.1%) used a negative-oriented metaphor, further increasing at 12 months (266; 78.5%), when they used fewer neutral-/positive-oriented metaphors (*p* < 0.001). At the 6-month follow-up, only three individual variables (female gender, education, and experiencing symptoms at the COVID-19 onset) were significantly different across the possible metaphor orientation; at 12 months, no individual variables were significantly associated. This study suggests increasingly negative lived experiences over time and the need for personalized healthcare pathways to face the long-term traumatic consequences of COVID-19.

## 1. Introduction

Accumulating evidence indicates that the coronavirus disease 2019 (COVID-19) pandemic carries risks in terms of physical health and psychological health, triggering several consequences. In fact, increased psychological distress across the general population has been documented [1,2], along with a higher risk of relapse in those individuals with mental health issues before the pandemic outbreak [3,4]. In addition, several reasons for the distressing effects of COVID-19 have been proposed including, but not limited to, its rapid escalation towards a Public Health Emergency of International Concern (PHEIC) as declared by the World Health Organization (WHO) [5], the evidence of a potentially fatal outcome [6], media depictions of its impact worldwide [7], the unprecedented quarantine and restrictions imposed on all people [8], and the potentially long-lasting adverse biological implications and consequences of the disease on mental health among surviving patients [9].

For all these reasons, the COVID-19 pandemic has represented a collective traumatic experience [10] with detrimental social, economic, and health consequences [11] whose impact should continue to be documented. Specifically, on a par with other national and international events carrying a collective trauma, such as wars [12] and natural disasters [13], there is a need to explore how the COVID-19 pandemic is affecting individuals’ ability to cope and be resilient in the face of what is a persisting collective trauma experience [11]. However, some caveats have been highlighted when attempting to document emotional responses to a traumatic event at the group level. As most events are rapid and unexpected, the available evidence is generally based on retrospective studies, which are susceptible to recall bias. Therefore, prospective evidence is required to document collective emotions as lived in reasonable temporal proximity to the traumatic event [11]. In addition, spontaneous emotions are often difficult to elicit due to a stigma-related unwillingness to disclose personal feelings [14]. In this regard, a line of research supports the usefulness of tropes and metaphors to overcome this difficulty and allow individuals to express their deep emotions related to being exposed to a traumatic event [11,15]. In particular, evidence indicates that metaphors may offer a bridge into the bereaved individual’s world of meaning, which can deepen understanding of their unique experience of suffering [15]. In addition, metaphors have been suggested to be particularly helpful in assessing emotional responses to emerging, unfamiliar, and still uncertain experiences [16]. This is particularly relevant to COVID-19, where evidence is continuously unfolding and is not always unequivocal [17].

Following on from previous evidence [11,16,17], this study was designed to describe COVID-19-related trauma and emotional processing among COVID-19 survivors at 6 and 12 months to provide information at clinical and public health levels. Specifically, the primary aim of the study was to collect ongoing, rather than retrospective, COVID-19 survivors’ pandemic-related experiences, using metaphors as a research tool to access the lived experiences of collective traumas. As a secondary aim, the study explored the socio-demographic variables, if any, associated with the emotional response of COVID-19 survivors as expressed through the metaphors.

## 2. Materials and Methods

### 2.1. Study Design

An observational follow-up survey, initiated in March/April 2020 and consisting of two time points, namely, 6 (October/November 2020) and 12 months after the COVID-19 onset (March/April 2021), was performed to collect qualitative variables [18]. The study design was chosen to obtain insights (a) from key informants on a not fully understood phenomenon [19], which COVID-19 is, (b) regarding their experience as survivors, (c) over time. According to the study design, the Strengthening the Reporting of Observational Studies in Epidemiology (STROBE) guidelines [20] were adopted to report methods and findings (see Supplementary Table S1).

### 2.2. Setting and Participants

**Figure 1:**
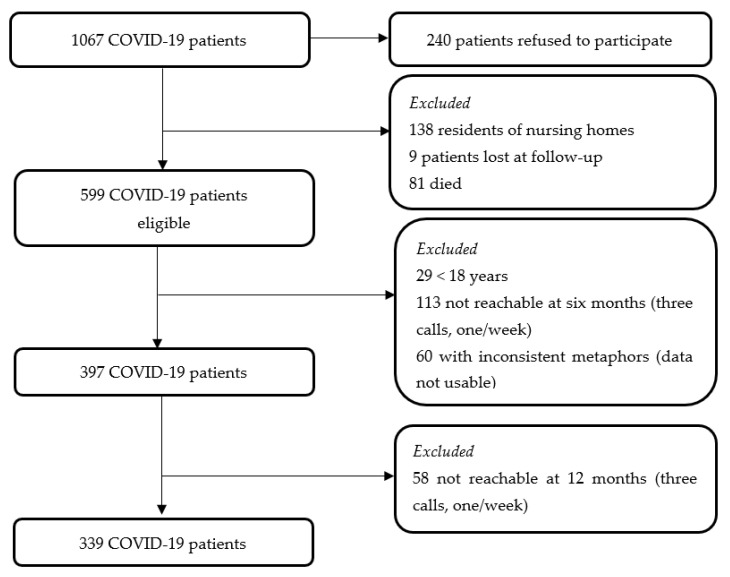
Flow chart of the COVID-19 patients interviewed at 6- and 12-month follow-ups. COVID-19, Coronavirus Disease 2019.

The study was performed in a reference infectious disease unit of a large academic hospital (>1000 beds) in the Friuli Venezia-Giulia Region (Italy). The target population of this study were the 1067 COVID-19 patients who were cared for in the first wave. Among them, 240 patients refused to participate in the study, 138 residents of nursing homes were excluded, nine survivors were lost in the follow-up, and 81 died. Therefore, 599 survivors were eligible. At 6 and 12 months after their disease onset, the patients included were those who were (a) >18 years, (b) reachable by telephone, and (c) willing to participate in telephone interviews. A total of 339 individuals participated in both the 6- and 12-month follow-up assessment, as reported in Figure 1.

For each eligible patient, data were collected at three time points: (1) at COVID-19 diagnosis, (2) after 6 and (3) after 12 months. At the disease onset, (a) socio-demographic (e.g., gender) and (b) clinical data (e.g., the severity of COVID-19) were all collected and recorded in a database. The severity of the COVID-19 disease was categorized as: asymptomatic; mild disease (without pneumonia); moderate disease (pneumonia); severe disease (severe pneumonia); or critical disease, including acute respiratory distress syndrome (ARDS), sepsis, and/or septic shock [21]. In addition, dyspnea at the onset was considered relevant in the context of COVID-19 disease and emotional impact [22]. For this reason, it was also registered.

### 2.3. Data Collection Instrument and Method

At the 6-month follow-up, an interview based on open- and closed-ended questions was conducted to investigate (a) the source of the contagion (e.g., relatives), (b) the subjective perception of being healed or not and, if any, the persisting symptoms, and (c) a metaphor or a word summarizing the whole COVID-19 experience up to that moment. Each participant was left free to share a symbolic summary of her/his experience by using a metaphor or a word condensing a meaning. Metaphors have been suggested to be effective in summarizing a phenomenon not previously documented [23], the meaning of complex realities [24], and that of lived experiences [25] based on their capacity to trigger an identification and categorization process through a linguistic device [26]. As a figure of speech, a metaphor might be considered the outcome of an experience but also as a ‘powerful metaphor [that] initiates and guides social processes’ [27].

At the 12-month follow-up, the same interview was repeated. Data were collected regarding (a) the perception of being healed and, if any, the persisting symptoms and (b) a metaphor or a word summarizing the entire COVID-19 experience up to the moment of the interview. A pilot phase assessing the interview guide feasibility, clarity, and understandability was performed involving ten patients and no changes were suggested.

### 2.4. Data Analysis

Collected data were first analyzed by calculating frequencies, percentages, averages, and confidence intervals (CI) at 95%. The following steps were considered to extract information from participants’ metaphors/words regarding the entire COVID-19 experience (hereinafter, metaphors). First, metaphors were: (a) reduced to labels to epitomize the meaning of the given experience in line with evidence supporting their effectiveness in summarizing a range of meanings [24]; (b) merged into their main orientations (positive, negative, or neutral) depending on the prevailing emotion embodied in the metaphor expressed, thus enhancing their fundamental role in representing the lived experience of patients; and (c) counted to compare the frequency of specific meanings [28] across different demographic and clinical profiles of patients, and over time. Then, the metaphors were processed by:

- Selection: when participants expressed more than one metaphor, only one was considered according to its intensity and capacity to condense the meaning of the whole experience as judged by two researchers; first, independently and then decided upon agreement.
- Summarization: the metaphors were then summarized in a single word expressing the ‘metaphor vehicle’ as a paradigmatic example of a given category [25] by conducting a content analysis [29]. The qualities of each metaphor were checked: some were left in their original structure (e.g., ‘like in a jail’), and others were left as expressing a process (e.g., ‘thinking’) or a feeling (e.g., ‘fear’). Considering that all expressions reflected how patients categorize and make sense of their lived experience, all were considered able to express the quality required by a metaphor [25].
- Categorization [28]: all metaphors were categorized into their emotional orientation (positive, neutral, or negative) according to the context of the expression reported by each participant as judged by two researchers; first, independently and then decided upon agreement.
- Analysis and comparison: a descriptive analysis of the metaphors was then performed according to their emotional orientation (positive, negative, or neutral) [30] across the main demographic and clinical variables and over time (at the 6- and 12-month follow-ups). Differences at the 6- and 12-month follow-ups and over time were explored using R software [31]. Testing procedures considered the Chi-squared test, the McNemar–Bowker symmetry test for categorical variables, and the single sample t-test for continuous variables [32]. The Kruskal–Wallis non-parametric testing procedure was used for numerical variables [33].

### 2.5. Rigour in Data Collection and Analysis

Three registered nurses educated to an advanced level (PhD and Master of Science in Nursing) with experience in qualitative research methods performed the data collection via telephone at both the 6- and 12-month follow-ups. Each patient was contacted up to three times/week, after which non-response was established as an exclusion criterion to prevent excessive intrusion. Preliminarily, the interviewers informed each participant again regarding the study aims and allowed each patient the freedom to establish whether and when to perform the interview according to his/her preferences. Patients were allowed to answer in their own words, and metaphors were recorded in a database and then repeated by the interviewer at the end of the call to ensure accuracy. Moreover, patients who reported persistent symptoms or issues were referred to the infectious disease unit or the psychiatric unit for consultation.

Rigor in data analysis was ensured by using the following strategies. First, three researchers worked on the metaphors independently in each step and agreed upon the findings to ensure investigator triangulation of the data analysis [34]. Second, given the novelty of the disease, researchers shared no preconceptions before either the data collection or the analysis. Third, selection bias [35] was considered by comparing the main individual variables between those involved and those excluded (see Supplementary Table S2). Fourth, given that the interview was performed in the Italian language, special attention was ensured during the translation process from the original language to English for publication purposes. In the process, two researchers were involved (MC, AP), who are experts in both languages and contexts, to prevent possible errors in interpretation. Moreover, they conducted the forward translation (from Italian to English) according to the World Health Organization’s guidelines [36].

## 3. Results

### 3.1. Patient Profile

There were 339 patients involved; the majority were female (176; 51.9%); the mean age was 52.9 years (CI 95% 51.2–54.6). Most of them were Italian (315; 92.9%) and were educated to secondary school level (160; 47.2%) or above (bachelor’s degree = 85; 25.1%; PhD = 3; 0.9%). At the onset, they were mainly diagnosed with mild COVID-19 disease (228; 67.3%), and slightly more than a quarter were hospitalized (93; 27.4%) for an average of 10.5 days (CI 95% 8.2–12.8). The majority reported previous comorbidities (176; 51.9%) and one or more symptoms at the COVID-19 onset (307; 90.6%). After 6 months, 115 patients (33.9%) reported persisting COVID-19 symptoms, a percentage that increased at 12 months (147; 43.4%) (*p* < 0.001) (Table 1).

### 3.2. Metaphors

#### 3.2.1. Metaphor Orientation at 6- and 12- Month Follow-Ups

All participants were able to express one or more metaphors to summarize their experience as COVID-19 survivors. Among the negative-oriented metaphors, “Fear”, “Nightmare”, “Bad..bad”, “Isolation”, “Traumatic”, “Concern”, “Like in a Jail”, “Destructive”, and “Terrible” were more often reported at both 6 and 12 months; on the other hand, “Rediscovery (Myself)”, “Thinking (an occasion of)”, and “Lucky” were mostly reported among the positive-oriented metaphors at both 6 and 12 months. Finally, among the neutral-oriented metaphors, “Surreal” prevailed at the 6-month follow-up, and “Indifference” was the most reported metaphor at the 12-month follow-up. A detailed presentation of all metaphors is reported in Supplementary Table S3.

As summarized in Table 2, at the 6-month follow-up, most participants (214; 63.1%) used a negative-oriented metaphor, 83 (24.5%) used a neutral-orientated metaphor and 42 (12.4%) used a positive-oriented metaphor. However, at the 12-month follow-up, more patients reported a negative-oriented metaphor (266; 78.5%) and less reported a neutral- (41; 12.1%) or positive-oriented metaphor (32; 9.4%). Moreover, as reported in Table 2, most people reporting a negative-oriented metaphor at the 6-month follow-up confirmed a negative-oriented one at the 12-month follow-up (190; 88.8%); a few changed their orientation either into a positive (11; 5.1%) or a neutral (13; 6.1%) metaphor. The majority of those using a neutral-oriented metaphor at the 6-month follow-up changed to a negative one at the 12-month follow-up (53; 63.9%). Similarly, most of those who used a positive-oriented metaphor at the 6-month follow-up expressed their lived experience by using a negative-oriented metaphor at the 12-month follow-up (23; 54.8%). As a result, the asymmetry in the changes observed in metaphor orientation between the 6- and 12-month follow-ups showed a worsening trend that was statistically significant (*p* < 0.001).

#### 3.2.2. Individual Variables and Metaphor Orientation at 6- and 12-Month Follow-Ups

At the 6-month follow-up, only three individual variables were associated significantly with metaphor orientation. Female patients more often expressed a negative-oriented metaphor (128; 59.8%) as compared to males who expressed mostly neutral- (53; 63.9%) and positive- (24; 57.1%) metaphors (*p* < 0.001). Those with middle school and bachelor level education (47; 21.9%; and 53; 24.8%; respectively) reported negative-oriented metaphors less often and neutral- (12; 41.5%; and 17; 20.5%; respectively) or positive-oriented metaphors more often (8; 19%; and 15; 35.7%; respectively) (*p* = 0.046). All patients reporting a positive-oriented metaphor (42; 100%) presented symptoms at the onset; whereas, a significantly lower proportion was symptomatic among those who summarized their experience as neutral (72; 86.7%) or negative (193; 90.2%) (*p* = 0.049). No statistical differences were found across the three different metaphor orientations in the other individual variables at the 12-month follow-up (Table 3).

## 4. Discussion

A few studies to date have investigated the pandemic experience by using the metaphor as a data collection tool. Available studies have investigated metaphors as expressed by individuals in Turkey [37] and in the US [11] during the COVID-19 pandemic; by family caregivers regarding their caregiving and bereavement in the context of the COVID-19 pandemic in Quebec [38]; or by COVID-19 survivors just after their experience in China [39]. Moreover, metaphors have been investigated while communicating issues related to the pandemic [40] as well as those used at societal and politics levels [41]. However, to the best of our knowledge, this is the first investigation that prospectively performed a comprehensive assessment of COVID-19-related trauma and emotion processing in COVID-19 first wave survivors by employing metaphors expressed at 6 and 12 months. Research evidence from this study suggests that most patients who have suffered from COVID-19 use a negative metaphor to describe their experience at 6 months. Further, findings indicate a worsening trend up to the 12-month follow-up, with a proportion of COVID-19 survivors reporting a negative connotation with their experience that was higher than the one collected at 6 months after the COVID-19 infection. Finally, male and less-educated patients as well as those who had symptoms at the onset were less likely to attribute a negative valence to their lived experience at 6 months, with such differences being lost at the 12-month follow-up where no individual variables emerged as being associated with metaphor orientation.

When it comes to psychological distress emerging after exposure to a traumatic stressor [42], a long-lasting debate regards the temporal stability or change in the presence of such distress. Early cross-sectional studies have certainly shown that distress can persist for up to several decades following various traumatic events, such as military combat [43], prison and war confinement [44], the Holocaust [45], natural disasters [46], and accidents [47]. However, due to their methodological design, these studies could not disentangle whether stress symptoms increase, reduce, or remain stable over time. As a result, two influential but contrasting stress models have been historically proposed regarding this issue. On the one hand, the stress-evaporation theory postulates that stress symptoms will abate over time in the absence of other psychological vulnerabilities. On the other hand, the residual stress hypothesis posits that traumatic stressors can produce chronic and long-lasting effects, even in individuals with good pre-traumatic adjustments [48]. Some studies of non-illness-related traumatic events [48] and life-threatening diseases, such as cancer [49], offer insights into such a debate. They conclude that neither previous stress disorder behaviors explain the persistence of distressing symptoms in the longer-term [48] nor is there a general diminishing of stress symptoms with time [49]. On the contrary, evidence from oncological studies indicates the possibility of a delayed onset of distress in some individuals [49], along with fluctuations with a waxing and waning symptom course, depending on individual characteristics, thus supporting the residual stress hypothesis. The current prospective study confirms and extends such knowledge, as it indirectly suggests that residual stress symptoms increase over time, rather than blunt, also in COVID-19 survivors. Further, significant fluctuations in connoting the COVID-19 experience negatively were also observed as a function of both COVID-19 survivors’ socio-demographic (i.e., gender and education) and clinical characteristics (COVID-19 being symptomatic or not at the onset).

In terms of predictors of negative metaphor attribution to the COVID-19-related experience, gender appeared to play a significant role, with female patients being more likely to report a negative valence at the 6-month follow-up. Independent research evidence would explain a higher level of negative emotions among women due to their ability to use a broader and more elaborate range of emotions than men [50,51,52]. Thus, findings from this study would not necessarily indicate greater distress in the female population after the beginning of the pandemic but confirm the female tendency to attribute greater severity to events in the context of the COVID-19 experience. Furthermore, this data interpretation seems to be corroborated by a counterbalanced trend at the 12-month follow-up assessment, with fewer women and more men using a negative metaphor, such that the gender gap in metaphor orientation is no longer significant. Overall, this finding carries public health implications, as it highlights a gender-driven difference in the timing of the emotional response to stress in COVID-19 survivors, which occurs earlier in female patients and later in male ones.

This study provides empirical evidence that education status may also be helpful in the epidemiological characterization of COVID-19-related negative emotion manifestation, with COVID-19 survivors performing differently in terms of metaphor orientation over the follow-up assessments. Specifically, those who had reached only the middle school educational stage were more likely to use neutral and positive metaphors at the six-month follow-up, but this trend was dramatically reduced in the longer term, with most patients adopting negative metaphors at the 12-month follow-up. In contrast, patients with higher educational attainment were substantially stable, showing a balanced distribution in terms of metaphor orientation over the two time points. Finally, patients with a bachelor’s degree, who had shown a preference for positive metaphors at the six-month follow-up, maintained this approach at the subsequent follow-up. Such a gradient of metaphor orientation over time is in line with evidence for higher well-being in adults with higher education [53]. Interestingly, such a positive relationship has been reported to be mediated by the higher degree of psychosocial and interpersonal resources among people with higher levels of education that would make them more able to access social support [54,55] and employ coping resources and strategies [56,57] as well as problem-solving and cognitive abilities [58] to handle stressful situations such as COVID-19.

Notably, patients that developed a symptomatic form of COVID-19 were more likely to use a positive metaphor at the 6-month follow-up. No significant differences were observed between symptomatic and asymptomatic COVID-19 patients in metaphor orientation at the 12-month follow-up. Although such findings may seem counterintuitive, research evidence suggests that psychological distress among asymptomatic patients may have been initially overlooked [59], resulting in levels that were higher than expected [60] and not inferior to those observed among symptomatic patients [61,62]. Several reasons for such elevated distress among asymptomatic patients have been proposed, including the psychological consequences of being quarantined [63,64] and the stigma-mediated distress [59] of representing the primary source of infection during the pandemic [65]. In addition, concern about being a possible asymptomatic carrier would be greater than being positive for COVID-19 [66]. One year after being infected, symptomatic status no longer predicted the emotional representation of being a COVID-19 survivor.

### Study Limitations

This study has several limitations. First, although not differing in some individual variables (e.g., gender and hospitalization), participants were significantly younger (52.9 years) and more often presenting with mild/moderate disease (67.3% and 16.5%, respectively) as compared to those not interviewed (58.9 years, 39.4% and 10.7% respectively), indicating a possible selection bias. Therefore, these findings are not generalizable to older patients with more severe forms of the disease. Second, the metaphors are culturally influenced, making the current findings not generalizable to other cultures. In addition, translation into another language may change the meaning of the metaphors [67]. Despite the adoption of strategies suggested in the available literature to ensure rigor [36], metaphors are associated with indirectness [67] and are thus difficult to translate, which calls for external validity of the findings. Third, according to the population-based study design adopted and the context of the COVID-19 pandemic as a collective trauma [11], all patients were involved independently of their asymptomatic or symptomatic presentation of the disease at the onset which might have influenced the findings. Fourth, in the absence of specific psychometric assessments of well-being and quality of life, no conclusions regarding more comprehensive psychosocial adjustment of COVID-19 survivors can be inferred from the metaphor orientations that emerged at 6 and 12 months and their changes over time. Fifth, only some bivariate analyses have been performed in line with the study’s secondary aims; therefore, a limited set of associations were explored without controlling for other potentially influential variables. In fact, additional environmental and personal factors (e.g., the employment status of participants before and after the disease, the possible financial difficulties that occurred, and the consequences of COVID-19 for the family) may have influenced the perceived experiences of the survivors, suggesting that further comprehensive studies in the field are required.

## 5. Conclusions

In conclusion, this study is suggestive of the evolving emotional status of COVID-19 survivors, with an increasingly negative lived experience over time. Furthermore, patients’ perceptions at 6 months after the acute phase were associated with socio-demographic variables, such as gender and education, and some clinical factors, such as being symptomatic at the onset. At the same time, any differences smoothed out in the longer term, at 12 months. The research findings may have important public health implications as they suggest the need for personalized healthcare pathways to tackle the potentially long-term traumatic consequences of COVID-19.

## Figures and Tables

**Table 1:** Main characteristics of interviewed patients.

At COVID-19 Onset	*n* = 339 (%)
*Gender* Female Male	176 (51.9) 163 (48.1)
*Age* (years), mean (CI 95%)	52.9 (51.2–54.6)
*Nationality* Italian Non-Italian	315 (92.9) 24 (7.1)
*Education* None Primary School Middle School Secondary School Bachelor’s Degree PhD	1 (0.3) 23 (6.8) 67 (19.8) 160 (47.2) 85 (25.1) 3 (0.9)
*Infected by whom* I don’t know Family members Colleagues Both	103 (30.4) 201 (59.3) 28 (8.2) 7 (2.1)
*COVID-19 severity, WHO scale* Asymptomatic Mild disease (without pneumonia) Moderate disease (pneumonia) Severe disease (severe pneumonia) Critical disease, including acute respiratory distress syndrome, sepsis and/or septic shock Missing	33 (9.7) 228 (67.3) 56 (16.5) 12 (3.5) 8 (2.4) 2 (0.6)
*Hospitalized for COVID-19*	93 (27.4)
*Duration of hospitalization* (days), mean (CI 95%)	10.5 (8.2–12.8)
*Previous comorbidities*	176 (51.9)
*COVID-19 symptoms at onset* Yes No	307 (90.6) 32 (9.4)
**At 6 months after the COVID-19 onset**	
*Persisting COVID-19 symptoms* ¥ Yes No Uncertain	115 (33.9) 218 (64.3) 6 (1.8)
**At 12 months after the COVID-19 onset**	
*Persisting COVID-19 symptoms* ¥ Yes No Uncertain	147 (43.4) 170 (50.1) 22 (6.5)

¥ *p* < 0.001 Chi-squared with bootstrap; CI, Confidence Interval; COVID-19, coronavirus disease 2019; *n*, Number; PhD, Doctor of Philosophy; SD, Standard Deviation; WHO, World Health Organization.

**Table 2:** Changes in the orientation of metaphors between the 6- and 12-month follow-ups.

At 6 Months—Metaphor Orientation, *n* (%)		At 12 Months—Metaphor Orientation, *n* (%)						*p*-Value °
		Negative 266 (78.5)		Neutral 41 (12.1)		Positive 32 (9.4)		
Negative Neutral Positive	214 (63.1) 83 (24.5) 42 (12.4)	Unchanged Worsened Worsened	190 (88.8) 53 (63.9) 23 (54.8)	Improved Unchanged Worsened	13 (6.1) 19 (22.9) 9 (21.4)	Improved Improved Unchanged	11 (5.1) 11 (13.2) 10 (23.8)	<0.001

° McNemar–Bowker symmetry test was applied to the 3 × 3 table.

**Table 3:** Metaphor orientation, according to patients’ characteristics (*n* = 339).

Variables and Time	Negative-Oriented		Neutral-Oriented		Positive-Oriented		*p*-Value	
At the COVID-19 Onset	6 Months 214 (%)	12 Months 266 (%)	6 Months 83 (%)	12 Months 41 (%)	6 Months 42 (%)	12 Months 32 (%)	6 Months	12 Months
*Gender °* Female Male	128 (59.8) 86 (40.2)	145 (54.5) 121 (45.5)	30 (36.1) 53 (63.9)	16 (39.0) 25 (61.0)	18 (42.9) 24 (57.1)	15 (46.9) 17 (53.1)	<0.001	0.152
*Age* (years), mean (CI 95%) °°	53.5 (51.5–55.6)	52.6 (50.7–54.5)	48.9 (45.3–52.5)	53.6 (48.1–59.1)	53.6 (49.1–58.1)	54.1 (48.5–59.7)	0.107	0.766
*Nationality* ° Italian Not Italian	197 (92.1) 17 (7.9)	246 (92.5) 20 (7.5)	78 (94.0) 5 (6.0)	39 (95.1) 2 (4.9)	40 (95.2) 2 (4.8)	30 (93.8) 2 (6.2)	0.696	0.874
*Education °* None Primary School Middle School Secondary School Bachelor’s Degree PhD	1 (0.5) 15 (7.0) 47 (21.9) 98 (45.8) 53 (24.8) 0 (-)	1 (0.4) 17 (6.4) 59 (22.2) 124 (46.6) 63 (23.7) 2 (0.7)	0 (-) 8 (9.6) 12 (41.5) 43 (51.8) 17 (20.5) 3 (3.6)	0 (-) 4 (9.8) 5 (12.2) 23 (56.1) 8 (19.5) 1 (2.4)	0 (-) 0 (-) 8 (19.0) 19 (45.2) 15 (35.7) 0 (-)	0 (-) 2 (6.3) 3 (9.4) 13 (40.6) 14 (43.7) 0 (-)	0.046	0.301
*Severity of COVID-19 disease, WHO scale °* Asymptomatic Mild disease Moderate disease Severe disease Critical disease Missing	21 (9.8) 142 (66.4) 36 (16.8) 7 (3.3) 7 (3.3) 1 (0.4)	23 (8.6) 181 (68.0) 47 (17.7) 6 (2.3) 7 (2.6) 2 (0.8)	8 (9.6) 60 (72.3) 14 (16.9) 1 (1.2) 0 (-) 0 (-)	8 (19.5) 28 (68.3) 3 (7.3) 2 (4.9) 0 (-) 0 (-)	4 (9.5) 26 (61.9) 6 (14.3) 4 (9.5) 1 (2.4) 1 (2.4)	2 (6.2) 19 (59.4) 6 (18.8) 4 (12.5) 1 (3.1) 0 (-)	0.432	0.161
*Hospitalized for COVID-19 °*	61 (28.5)	71 (26.7)	20 (24.1)	9 (22.0)	12 (28.6)	13 (40.6)	0.735	0.175
*Duration of hospitalization* (days), mean (CI 95%) °°	10.3 (7.9–12.6)	9.7 (7.4–12.0)	7.6 (2.3–12.9)	9.1 (2.4–15.8)	14.1 (4.4–23.8)	13.8 (2.6–25.0)	0.596	0.079
*Infected by whom °* I don’t know Family members Colleagues Both	63 (29.5) 133 (62.1) 15 (7.0) 3 (1.4)	78 (29.3) 157 (59.0) 25 (9.4) 6 (2.3)	29 (34.9) 46 (55.5) 7 (8.4) 1 (1.2)	13 (31.7) 28 (68.3) 0 (-) 0 (-)	11 (26.2) 22 (52.4) 6 (14.3) 3 (7.1)	12 (37.5) 16 (50.0) 3 (9.4) 1 (3.1)	0.134	0.351
*COVID-19 symptoms at the onset °* Yes	193 (90.2)	244 (91.7)	72 (86.7)	34 (82.9)	42 (100)	29 (90.6)	0.049	0.207
*Comorbidities*	115 (53.7)	135 (50.8)	42 (50.6)	22 (53.7)	19 (45.2)	19 (59.4)	0.575	0.661
**At 6 and at 12 months after the COVID-19 onset**								
*Feeling healed at the time of the interview ° * Yes No/Uncertain	156 (72.9) 58 (27.1)	196 (73.7) 70 (26.3)	66 (79.5) 17 (20.5)	35 (85.4) 6 (14.6)	32 (76.2) 10 (23.8)	23 (71.9) 9 (28.1)	0.417	0.273
*Persisting COVID-19 symptoms °* Yes No/Uncertain	75 (35.0) 139 (65.0)	125 (47.0) 141 (53.0)	28 (33.7) 55 (66.3)	11 (26.8) 30 (73.2)	12 (28.6) 30 (71.4)	11 (34.4) 21 (65.6)	0.488	0.351

° Chi-squared *p*-values obtained considering a simulation approach; °° Kruskal–Wallis. CI, Confidence Interval; COVID-19, Coronavirus Disease 2019; *n*, Number; PhD, Doctor of Philosophy; WHO, World Health Organization.

## Data Availability

Data available on request due to restrictions, e.g., privacy or ethical.

## Supplementary Materials

The following supporting information can be downloaded at: https://www.mdpi.com/article/10.3390/ijerph20021390/s1, Table S1: Strengthening the reporting of observational studies in epidemiology (STROBE) Statement-Cohort studies; Table S2: Patients interviewed, not interviewed, and died, main profile. Table S3: ‘My lived experience as a COVID-19 survived patient’: metaphor orientation.

## References

[1] Lima C.K.T., Carvalho P.M.M., Lima I.A.A.S., Nunes J.V.A.O., Saraiva J.S., de Souza R.I., da Silva C.G.L., Neto M.L.R. (2020). The emotional impact of Coronavirus 2019-nCoV (new Coronavirus disease). Psychiatry Res..

[2] Pigaiani Y., Zoccante L., Zocca A., Arzenton A., Menegolli M., Fadel S., Ruggeri M., Colizzi M. (2020). Adolescent Lifestyle Behaviors, Coping Strategies and Subjective Wellbeing during the COVID-19 Pandemic: An Online Student Survey. Healthcare.

[3] Yao H., Chen J.H., Xu Y.F. (2020). Patients with mental health disorders in the COVID-19 epidemic. Lancet Psychiatry.

[4] Colizzi M., Sironi E., Antonini F., Ciceri M.L., Bovo C., Zoccante L. (2020). Psychosocial and Behavioral Impact of COVID-19 in Autism Spectrum Disorder: An Online Parent Survey. Brain Sci..

[5] Cucinotta D., Vanelli M. (2020). WHO Declares COVID-19 a Pandemic. Acta Biomed..

[6] Onder G., Rezza G., Brusaferro S. (2020). Case-Fatality Rate and Characteristics of Patients Dying in Relation to COVID-19 in Italy. JAMA.

[7] Garfin D.R., Silver R.C., Holman E.A. (2020). The novel coronavirus (COVID-2019) outbreak: Amplification of public health consequences by media exposure. Health Psychol..

[8] Wilder-Smith A., Freedman D.O. (2020). Isolation, quarantine, social distancing and community containment: Pivotal role for old-style public health measures in the novel coronavirus (2019-nCoV) outbreak. J. Travel Med..

[9] Colizzi M., Peghin M., De Martino M., Bontempo G., Gerussi V., Palese A., Isola M., Tascini C., Balestrieri M. (2022). Mental health symptoms one year after acute COVID-19 infection: Prevalence and risk factors. Rev. Psiquiatr. Salud Ment..

[10] Hirschberger G. (2018). Collective Trauma and the Social Construction of Meaning. Front. Psychol..

[11] Stanley B.L., Zanin A.C., Avalos B.L., Tracy S.J., Town S. (2021). Collective Emotion During Collective Trauma: A Metaphor Analysis of the COVID-19 Pandemic. Qual. Health Res..

[12] Garcia D., Rimé B. (2019). Collective Emotions and Social Resilience in the Digital Traces After a Terrorist Attack. Psychol. Sci..

[13] Richardson B., Maninger L. (2016). “We Were All in the Same Boat”: An Exploratory Study of Communal Coping in Disaster Recovery. South. Commun. J..

[14] Hatzenbuehler M.L., Nolen-Hoeksema S., Dovidio J. (2009). How does stigma “get under the skin”?: The mediating role of emotion regulation. Psychol. Sci..

[15] Neimeyer R. (1999). Narrative strategies in grief therapy. J. Constr. Psychol..

[16] Clair R. (1993). The Use of Framing Devices to Sequester Organizational Narratives—Hegemony and Harassment. Commun. Monogr..

[17] Escandón K., Rasmussen A.L., Bogoch I.I., Murray E.J., Popescu S.V., Kindrachuk J. (2021). COVID-19 false dichotomies and a comprehensive review of the evidence regarding public health, COVID-19 symptomatology, SARS-CoV-2 transmission, mask wearing, and reinfection. BMC Infect. Dis..

[18] Kingsley C., Patel S. (2017). Patient-reported outcome measures and patient-reported experience measures. BJA Educ..

[19] Sandelowski M. (2000). Whatever happened to qualitative description?. Res. Nurs. Health.

[20] von Elm E., Altman D.G., Egger M., Pocock S.J., Gøtzsche P.C., Vandenbroucke J.P. (2007). The Strengthening the Reporting of Observational Studies in Epidemiology (STROBE) statement: Guidelines for reporting observational studies. Ann. Intern. Med..

[21] World Health Organization (2021). Living Guidance for Clinical Management of COVID-19: Living Guidance.

[22] Schmier J.K., Halpern M.T., Higashi M.K., Bakst A. (2005). The quality of life impact of acute exacerbations of chronic bronchitis (AECB): A literature review. Qual. Life Res..

[23] Lakoff G., Johnson M. (2008). Metaphors We Live by.

[24] Miles M.B., Huberman A.M. (1994). Qualitative Data Analysis: An Expanded Sourcebook.

[25] Gibbs R.W. (1992). Categorization and metaphor understanding. Psychol. Rev..

[26] Rodríguez C., Bélanger E. (2014). Stories and metaphors in the sensemaking of multiple primary health care organizational identities. BMC Fam. Pract..

[27] Czarniawska-Joerges B. (1994). Narratives of Individual and Organizational Identities. Ann. Int. Commun. Assoc..

[28] Steger T. (2007). The Stories Metaphors Tell: Metaphors as a Tool to Decipher Tacit Aspects in Narratives. Field Methods.

[29] Vaismoradi M., Turunen H., Bondas T. (2013). Content analysis and thematic analysis: Implications for conducting a qualitative descriptive study. Nurs. Health Sci..

[30] Ambrosi E., Canzan F. (2013). Introduction to qualitative research: The main approaches and designs. Assist. Inferm. Ric..

[31] R Core Team (2021). R: A Language and Environment for Statistical Computing.

[32] Agresti A. (2003). Categorical Data Analysis.

[33] Hollander M., Wolfe D.A. (1973). Nonparametric Statistical Methods.

[34] Carter N., Bryant-Lukosius D., DiCenso A., Blythe J., Neville A.J. (2014). The use of triangulation in qualitative research. Oncol. Nurs. Forum.

[35] Gerhard T. (2008). Bias: Considerations for research practice. Am. J. Health Syst. Pharm..

[36] World Health Organization Process of Translation and Adaptation of Instruments. https://www.coursehero.com/file/30372721/WHO-Process-of-translation-and-adaptation-of-instrumentspdf/.

[37] Gök A., Kara A. (2022). Individuals’ conceptions of COVID-19 pandemic through metaphor analysis. Curr. Psychol..

[38] Guité-Verret A., Vachon M., Ummel D., Lessard E., Francoeur-Carron C. (2021). Expressing grief through metaphors: Family caregivers’ experience of care and grief during the COVID-19 pandemic. Int. J. Qual. Stud. Health Well-Being.

[39] Deng Y., Yang J., Wan W. (2021). Embodied metaphor in communication about lived experiences of the COVID-19 pandemic in Wuhan, China. PLoS ONE.

[40] Semino E. (2021). “Not Soldiers but Fire-fighters”—Metaphors and COVID-19. Health Commun..

[41] Rahman S.Y. (2020). ‘Social distancing’ during COVID-19: The metaphors and politics of pandemic response in India. Health Sociol. Rev..

[42] American Psychiatric Association (2013). Diagnostic and Statistical Manual of Mental Disorders, Fifth Edition (DSM-5).

[43] Archibald H.C., Tuddenham R.D. (1965). Persistent Stress Reaction After Combat: A 20 Year Follow-Up. Arch. Gen. Psychiatry.

[44] Goldstein G., van Kammen W., Shelly C., Miller D.J., van Kammen D.P. (1987). Survivors of imprisonment in the Pacific theater during World War II. Am. J. Psychiatry.

[45] Eaton W.W., Sigal J.J., Weinfeld M. (1982). Impairment in Holocaust survivors after 33 years: Data from an unbiased community sample. Am. J. Psychiatry.

[46] Green B.L., Lindy J.D., Grace M.C., Gleser G.C., Leonard A.C., Korol M., Winget C. (1990). Buffalo Creek survivors in the second decade: Stability of stress symptoms. Am. J. Orthopsychiatry.

[47] Green B.L., Grace M.C., Gleser G.C. (1985). Identifying survivors at risk: Long-term impairment following the Beverly Hills Supper Club fire. J. Consult. Clin. Psychol..

[48] Watson C.G., Kucala T., Manifold V., Vassar P. (1989). Childhood stress disorder behaviors in veterans who do and do not develop posttraumatic stress disorder. J. Nerv. Ment. Dis..

[49] Andrykowski M.A., Cordova M.J., McGrath P.C., Sloan D.A., Kenady D.E. (2000). Stability and change in posttraumatic stress disorder symptoms following breast cancer treatment: A 1-year follow-up. Psychooncology.

[50] Seiffge-Krenke I. (1992). Coping behavior of Finnish adolescents: Remarks on a cross-cultural comparison. Scand. J. Psychol..

[51] Frydenberg E., Lewis R. (1991). Adolescent coping: The different ways in which boys and girls cope. J. Adolesc..

[52] Frydenberg E., Lewis R. (1993). Boys play sport and girls turn to others: Age, gender and ethnicity as determinants of coping. J. Adolesc..

[53] Raghupathi V., Raghupathi W. (2020). The influence of education on health: An empirical assessment of OECD countries for the period 1995–2015. Arch. Public Health.

[54] Berkman L.F., Syme S.L. (1979). Social networks, host resistance, and mortality: A nine-year follow-up study of Alameda County residents. Am. J. Epidemiol..

[55] Manton K.G., Corder L., Stallard E. (1997). Chronic disability trends in elderly United States populations: 1982–1994. Proc. Natl. Acad. Sci. USA.

[56] Folkman S., Lazarus R.S. (1980). An analysis of coping in a middle-aged community sample. J. Health Soc. Behav..

[57] Wheaton B. (1983). Stress, personal coping resources, and psychiatric symptoms: An investigation of interactive models. J. Health Soc. Behav..

[58] George L.K., Gwyther L.P. (1986). Caregiver well-being: A multidimensional examination of family caregivers of demented adults. Gerontologist.

[59] Chen H., Chen Y., Zhang Y., Wang Z., Shi D., Liu J., Yang X., Xu L., Cai Y., Hu F. (2022). Social Stigma and Depression among Asymptomatic COVID-19 Carriers in Shanghai, China: The Mediating Role of Entrapment and Decadence. Int. J. Environ. Res. Public Health.

[60] Upadhyay R., Sweta, Singh B., Singh U. (2020). Psychological impact of quarantine period on asymptomatic individuals with COVID-19. Soc. Sci. Humanit. Open.

[61] Deng J., Zhou F., Hou W., Silver Z., Wong C.Y., Chang O., Huang E., Zuo Q.K. (2021). The prevalence of depression, anxiety, and sleep disturbances in COVID-19 patients: A meta-analysis. Ann. N. Y. Acad. Sci..

[62] Ma Y.F., Li W., Deng H.B., Wang L., Wang Y., Wang P.H., Bo H.X., Cao J., Zhu L.Y., Yang Y. (2020). Prevalence of depression and its association with quality of life in clinically stable patients with COVID-19. J. Affect. Disord..

[63] Shigemura J., Ursano R.J., Morganstein J.C., Kurosawa M., Benedek D.M. (2020). Public responses to the novel 2019 coronavirus (2019-nCoV) in Japan: Mental health consequences and target populations. Psychiatry Clin. Neurosci..

[64] Wang C., Pan R., Wan X., Tan Y., Xu L., Ho C.S., Ho R.C. (2020). Immediate Psychological Responses and Associated Factors during the Initial Stage of the 2019 Coronavirus Disease (COVID-19) Epidemic among the General Population in China. Int. J. Environ. Res. Public Health.

[65] Bai Y., Yao L., Wei T., Tian F., Jin D.Y., Chen L., Wang M. (2020). Presumed Asymptomatic Carrier Transmission of COVID-19. JAMA.

[66] Germani A., Buratta L., Delvecchio E., Mazzeschi C. (2020). Emerging Adults and COVID-19: The Role of Individualism-Collectivism on Perceived Risks and Psychological Maladjustment. Int. J. Environ. Res. Public Health.

[67] Hegrenæs C. (2014). Conceptual Metaphors in Translation: A Corpus-based Study on Quantitative Differences between Translated and Non-Translated English. Metaphor and Intercultural Communication.

