# Supplementary material for: Using Metaphors to Understand Suffering in COVID-19 Survivors: A Two Time-Point Observational Follow-Up Study
Source: Int J Environ Res Public Health. 2023 Jan 12;20(2):1390. doi: 10.3390/ijerph20021390 (PMC9859410; doi:10.3390/ijerph20021390)
Supplement: Supplementary file 1 [file ijerph-20-01390-s001.zip › ijerph-2135943-supplementary.pdf]

Supplementary Table S1. Strengthening the reporting of observational studies in epidemiology (STROBE) Statement - Cohort studies [20]

	Item No	Recommendation	Section
**Title and abstract**	1	(*a*) Indicate the study's design with a commonly used term in the title or the abstract	Abstract and title
		(*b*) Provide in the abstract an informative and balanced summary of what was done and what was found	Abstract
**Introduction**			
Background/rationale	2	Explain the scientific background and rationale for the investigation being reported	Introduction
Objectives	3	State specific objectives, including any prespecified hypotheses	Introduction
Methods			
Study design	4	Present key elements of study design early in the paper	Methods<br>Study design
Setting	5	Describe the setting, locations, and relevant dates, including periods of recruitment, exposure, follow-up, and data collection	Setting and participants
Participants	6	(*a*) Give the eligibility criteria, and the sources and methods of selection of participants. Describe methods of follow-up	Setting and participants
		(*b*) For matched studies, give matching criteria and number of exposed and unexposed	Not appropriate
Variables	7	Clearly define all outcomes, exposures, predictors, potential confounders, and effect modifiers. Give diagnostic criteria, if applicable	Data Collection Instrument and Method
Data sources/ measurement	8 *	For each variable of interest, give sources of data and details of methods of assessment (measurement). Describe comparability of assessment methods if there is more than one group	Data Collection Instrument and Method
Bias	9	Describe any efforts to address potential sources of bias	Rigour in Data Collection and Analysis
Study size	10	Explain how the study size was arrived at	Patient profile,<br>Figure 1
Quantitative variables	11	Explain how quantitative variables were handled in the analyses. If applicable, describe which groupings were chosen and why	Data Collection Instrument and Method<br>Data Analysis
Statistical methods	12	(*a*) Describe all statistical methods, including those used to control for confounding	Data Analysis
		(*b*) Describe any methods used to examine subgroups and interactions	
		(*c*) Explain how missing data were addressed	
		(*d*) If applicable, explain how loss to follow-up was addressed	
		(*e*) Describe any sensitivity analyses	
**Results**			
Participants	13 *	(a) Report numbers of individuals at each stage of study—eg numbers potentially eligible, examined for eligibility, confirmed eligible, included in the study, completing follow-up, and analysed	Figure 1
		(b) Give reasons for non-participation at each stage	Figure 1
		(c) Consider use of a flow diagram	Figure 1

Descriptive data	14 *	(a) Give characteristics of study participants (eg demographic, clinical, social) and information on exposures and potential confounders	Table 1, Table 2, Table 3
		(b) Indicate number of participants with missing data for each variable of interest	Table 1, Table 2, Table 3
		(c) Summarise follow-up time (eg, average and total amount)	Table 1, Table 2, Table 3
Outcome data	15 *	Report numbers of outcome events or summary measures over time	Results<br>Table 1, Table 2, Table 3
Main results	16	(*a*) Give unadjusted estimates and, if applicable, confounder-adjusted estimates and their precision (eg, 95% confidence interval). Make clear which confounders were adjusted for and why they were included	Results<br>Table 1, Table 2, Table 3<br>Supplementary Table 3
		(*b*) Report category boundaries when continuous variables were categorized	
		(*c*) If relevant, consider translating estimates of relative risk into absolute risk for a meaningful time period	
Other analyses	17	Report other analyses done—eg analyses of subgroups and interactions, and sensitivity analyses	Supplementary Table 2
**Discussion**			
Key results	18	Summarise key results with reference to study objectives	Discussion
Limitations	19	Discuss limitations of the study, taking into account sources of potential bias or imprecision. Discuss both direction and magnitude of any potential bias	Discussion<br>Study limitations
Interpretation	20	Give a cautious overall interpretation of results considering objectives, limitations, multiplicity of analyses, results from similar studies, and other relevant evidence	Discussion
Generalisability	21	Discuss the generalisability (external validity) of the study results	Discussion/conclusions
**Other information**			
**Funding**	22	Give the source of funding and the role of the funders for the present study and, if applicable, for the original study on which the present article is based	None

* Give information separately for exposed and unexposed groups.

Supplementary Table S2. COVID-19 patients interviewed, not interviewed, and died: main profile.

Patients COVID-19 (N=1097)	Interviewed N= 339 (%)	Not Interviewed N= 647 (%)	Died N= 81 (%)	p-Value (Interviewed vs. Not Interviewed)
Gender				
Female	176 (51.9)	368 (56.9)	52 (64.2)	0.156 °
Male	163 (48.1)	279 (43.1)	29 (35.8)	
Age (years), mean (CI 95%)	52.9 (51.2-54.6)	58.9	83.2 (81.0-85.5)	<0.001 °°
WHO scale § for COVID-19 disease				<0.001 °°°
Asymptomatic	33 (9.7)	95 (14.7)	0 (-)	
Mild disease (without pneumonia)	228 (67.3)	255 (39.4)	3 (3.7)	
Moderate disease (pneumonia)	56 (16.5)	69 (10.7)	7 (8.6)	
Severe disease (severe pneumonia)	12 (3.5)	28 (4.3)	19 (23.5)	
Critical disease (acute respiratory distress syndrome, sepsis and/or septic shock	8 (2.4)	16 (2.5)	16 (19.7)	
Missing	2 (0.6)	184 (28.4)	36 (44.4)	
Hospitalized for COVID-19	93 (27.4)	143 (22.1)	81 (100)	0.074°

° Chi-squared test, °° single sample t-test for the mean (H0: mean age = 58.9), °°° Chi-squared test are computed omitting data regarding those patients who died, § WHO scale [21], asymptomatic; mild disease (without pneumonia); moderate disease (pneumonia); severe disease (severe pneumonia); critical disease, including acute respiratory distress syndrome (ARDS), sepsis and/or septic shock.

CI, Confidence Interval; COVID-19, Coronavirus Disease 2019; N, Number; WHO, World Health Organization.

Supplementary Table S3. *'My lived experience as a COVID-19 survived patient'*: metaphor orientation (N=339).

**Negative-Oriented**			**Neutral-Oriented**			**Positive-Oriented**		
**Metaphors, N (%)**	**6 months 214 (63.1)**	**12 months 266 (78.5)**	**Metaphors**	**6 months 83 (24.5)**	**12 months 41 (12.1)**	**Metaphors**	**6 months 42 (12.4)**	**12 months 32 (9.4)**
Fear	18	32	Surreal	12	2	Rediscovery (myself)	11	2
Nightmare	17	12	Strange	9	5	Thinking (an occasion of)	7	4
Bad…bad	16	30	Unexpected	8	1	Lucky	5	9
Upsetting	15	12	A test	8	0	More attention (to my-self)	4	2
Isolation	13	11	Change	6	0	Tranquillity	4	3
Traumatic	10	7	Acceptance	6	2	Positive	3	2
Concern	9	11	Indifference	5	19	Resilience	2	1
Like in jail	8	8	As a flu	5	5	Gifted time	2	1
Harsh	8	8	I don't know	5	1	Teaching	2	2
Difficult	8	5	Hoax	4	1	Serenity	1	4
Anguish	7	8	Insecurity	4	3	Rest	1	2
Tragic	6	7	New	4	1			
Drama	6	5	Uncertainty	2	1			
Negative	5	2	Indescribable	2	0			
Don't recommend it	5	1	Long	2	0			
Anxiety	4	6	Striking	1	0			
Heavy	3	4						
Scary	3	4						
Loneliness	3	4						

Terrible	3	10
Superficiality	3	0
Shock	3	2
Suffering	3	1
To forget	3	0
Abandonment	2	3
Devastating	2	6
Like dying	2	1
Nuisance	2	2
Sadness	2	2
Limiting	2	2
It leaves a mark	2	1
Impotence	2	1
Concern about family	2	0
A parenthesis	2	1
Stress	2	3
Fatigue	2	2
Destructive	2	11
Unforgettable	1	4
Discrimination	1	3
Chaos	1	5
Confusion	1	3
Impressive	1	0

Terrifying	1	0
Challenging	1	0
Distortion	1	0
Painful	1	0
Anger	0	1
Tiring	0	12
Alienating	0	7
Like an infector	0	3
Sneaky	0	3

COVID-19, Coronavirus Disease 2019; N, Number.
